# An ansatz for solving nonlinear partial differential equations in mathematical physics

**DOI:** 10.1186/s40064-015-1652-9

**Published:** 2016-01-07

**Authors:** M. Ali Akbar, Norhashidah Hj. Mohd. Ali

**Affiliations:** Department of Applied Mathematics, University of Rajshahi, Rajshahi, Bangladesh; School of Mathematical Sciences, Universiti Sains Malaysia, George Town, Malaysia

**Keywords:** Ansatz method, (1+1)-dimensional modified Benjamin–Bona–Mahony equation, Traveling wave solutions, NLPDEs, 35C07, 35C08, 35C09, 35D90, 35K90

## Abstract

In this article, we introduce an ansatz involving exact traveling wave solutions to nonlinear partial differential equations. To obtain wave solutions using direct method, the choice of an appropriate ansatz is of great importance. We apply this ansatz to examine new and further general traveling wave solutions to the (1+1)-dimensional modified Benjamin–Bona–Mahony equation. Abundant traveling wave solutions are derived including solitons, singular solitons, periodic solutions and general solitary wave solutions. The solutions emphasize the nobility of this ansatz in providing distinct solutions to various tangible phenomena in nonlinear science and engineering. The ansatz could be more efficient tool to deal with higher dimensional nonlinear evolution equations which frequently arise in many real world physical problems.

## Background

It is eminent that nonlinear partial differential equations (NLPDEs) are widely used as models to depict many important complex physical phenomena in a variety of fields of science and engineering, such as, nonlinear optics, solid state physics, plasma physics, chemical kinematics, fluid mechanics, chemistry, biology and many others. In the recent years, significant improvements have been made in searching wave solutions to NLPDEs. Several effective and powerful methods have been established to deal with NLPDEs. For instance, the Backlund transformation method (Rogers and Shadwick 1982), the inverse scattering method (Ablowitz and Clarkson 1991), the Darboux transformation method (Rogers and Schief 2003), the homogeneous balance method (Wang 1995; Wang et al. 1996), the tanh-function method (Parkes and Duffy 1996), the Jacobi elliptic function expansion method (Liu et al. 2001), the exp-function method (He and Wu 2006; Akbar and Ali 2011; Naher et al. 2012), the homotopy perturbation method (Jafari and Aminataei 2010; Yildirim et al. 2011), the Adomian decomposition method (Adomian 1994), the ansatz method (Bekir et al. 2012), the modified simple equation method (Jawad et al. 2010; Khan and Akbar 2013), etc.

A further significant method was introduced by Wang et al. (2008) for obtaining exact solutions to NLPDEs, called the (*G*^′^/*G*)-expansion method. The (*G*^′^/*G*)-expansion method is based on the assumptions that the wave solutions can be expressed by a polynomial in (*G*^′^/*G*), where the second order linear ordinary differential equation (ODE) *G*^ʺ^ + *λG*^′^ + *μG* = 0 has been executed as an auxiliary equation, *λ* and *μ* are arbitrary constants. The (*G*^′^/*G*)-expansion method is one of the powerful methods to investigate nonlinear problems. Thus, diverse group of scientists studied different kind of NLPDEs and constructed solitons, topological solitons, singular solitons and other kinds of general solitary wave solutions via this method. Applications of this method can be found in references, Akbar et al. (2012a, b, c), Feng and Zheng (2010), Feng et al. (2011) and the references therein for better understanding.

In order to establish the usefulness and reliability of the (*G*^′^/*G*)-expansion method and to enlarge the range of its pertinence, further research has been carried out by numerous researchers. For example, Zhang et al. (2010) presented an improved (*G*^′^/*G*)-expansion method to look into more general traveling wave solutions. In the original method, the traveling wave solutions were presented in the form $u(\xi )\; = \; \sum\nolimits_{i = 0}^{n} {a_{i} (G^{\prime } /G)^{i} }$ where *a*_*n*_ ≠ 0, while Zhang et al. (2010) assert that the solution can be expressed as $u(\xi )\; = \; \sum\nolimits_{i = - n}^{n} {a_{i} (G^{\prime } /G)^{i} ,}$ wherein *a*_−*n*_ or *a*_*n*_ could be zero, but both of them cannot be zero at the same time. Here, we observe that the range of the index *i* is from −*n* to *n* in place of 0 to *n* of the basic (*G*^′^/*G*)-expansion method. Thus, (*G*^′^/*G*)-expansion method established by Wang et al. (2008) is a particular case of the improved (*G*^′^/*G*)-expansion method. Li et al. (2010) presented a two-variable (*G*^′^/*G*, 1/*G*)-expansion method. They supposed that the solution to the NLEEs can be expressed in the form: $u(\xi )\; = \;\sum\nolimits_{i = 0}^{n} {a_{i} \varphi^{i} } + \sum\nolimits_{i = 0}^{n} {b_{i} \varphi^{i - 1} \psi }$, where *φ* = (*G*^′^/*G*), *ψ* = 1/*G* and *G* can be found from the linear differential equation $G^{\prime \prime } (\xi ) + \lambda G(\xi ) = \mu$. Zayed (2009) developed an alternative approach of the (*G*^′^/*G*)-expansion method, where *G*(*ξ*) satisfies the Jacobi elliptical equation $[G^{\prime } (\xi )]^{ 2} = e_{ 2} G^{ 4} (\xi ) + e_{ 1} G^{ 2} (\xi ) + e_{0}$. Zayed (2011) also developed a further alternative approach of this method wherein *G*(*ξ*) satisfies the Riccati equation $G^{\prime } (\xi ) = A + BG^{ 2} (\xi )$. Guo and Zhou (2010) established an extended (*G*^′^/*G*)-expansion. Akbar et al. (2012b) presented a generalized and improved (*G*^′^/*G*)-expansion method which provides further new solutions than the improved (*G*^′^/*G*)-expansion method (Zhang et al. 2010).

In the present article, we derive abundant traveling wave solutions to NLPDEs via the (1+1)-dimensional mBBM equation with some new solutions. To this end, a new ansatz involving the (*G*^′^/*G*)-expansion method will be presented. To depict the novelty, reliability and advantages, we compare the acquired solutions to those solutions attained by Zayed and Al-Joudi (2010). These solutions include soliton, topological soliton, singular soliton and generalized solitary wave solutions which might be helpful to analyze complex phenomena.

## Description of the method

In this section, we present the description of the method. We consider the general NLPDE in the following form

1 $$H(u,\,u_{t} ,\,\,u_{x} ,\,\,u_{t\,t} ,\,\,u_{t\,x} ,\,\,u_{x\,x} ) = 0,$$

where *u* is an unknown function which depends on *x* and *t* and *H* is a polynomial of *u* = *u* (*x*, *t*) and its partial derivatives in which linear term(s) of the highest order and highest order nonlinear term(s) exist. The most important steps of the new ansatz are as follows:

Step 1: In order to find traveling wave solutions of (1), we introduce the wave variable

2 $$u\;(x,t) = u\;(\xi ),\quad \xi = x - Vt,$$

where the constant *V* is the traveling wave speed to be determined. The traveling wave transformation (2) permits us to convert (1) into an ordinary differential equation (ODE) for *u* = *u*(*ξ*):

3 $$R\,(u,\, - V\,u^{{\prime }} ,\,V^{2} u^{\prime \prime } ,\,u^{\prime \prime } , \ldots ),$$

where in the superscripts denote the ordinary derivatives with respect to *ξ*.

Step 2: Integrate (3) term by term one or more times according to possibility that yields constant(s) of integration. The integration constant(s) may be zero for simplicity.

Step 3: We assume the traveling wave solution of (3) can be expressed in the following form:

4 $$u\;(\xi ) = \sum\limits_{i = - N}^{N} {\left\{ {\frac{{a_{i} \,(G^{{\prime }} /G)^{i} }}{{\left( {1 + \lambda \,(G^{{\prime }} /G)} \right)^{\,i} }} + b_{i} \,(G^{{\prime }} /G)^{i - 1} \sqrt {\sigma \,\left( {1 + \frac{1}{\mu }\,(G^{{\prime }} /G)^{2} } \right)} } \right\}} ,$$

where *G* = *G*(*ξ*) satisfies the auxiliary equation

5 $$G^{{\prime \prime }} + \mu \,G = 0.$$

*a*_*i*_, *b*_*i*_ (*i* = −*N*, …, *N*) and *λ* are constants to be determined, *σ* = ±1, *μ* ≠ 0 and *N* is a positive integer.

Step 4: To fix the positive integer *N*, we consider the homogeneous balance between the highest-order linear terms with the nonlinear terms of the highest order in (3).

Step 5: We substitute (4) into (3) including (5) and collect all terms of same powers of (*G*^′^/*G*)^*j*^ and $(G^{{\prime }} /G)^{j} \,\sqrt {\sigma \,[1 + (G^{{\prime }} /G)^{2} /\mu ]}$ together. Setting each coefficient to zero yields an over-determined system of algebraic equations. We solve the system of algebraic equations for *a*_*i*_, *b*_*i*_ (*i* = −*N*···*N*) and *λ*, *V*.

Step 6: The general solutions of (5) yield:

6 $$\begin{aligned} &F_{1} (\xi ) = \frac{{G^{\prime}}}{G} = \sqrt { - \mu } \left( {\frac{{A\,\sinh (\sqrt { - \mu } \,\xi ) + B\,\cosh (\sqrt { - \mu } \,\xi )}}{{A\,\cosh (\sqrt { - \mu } \,\xi ) + B\,\sinh (\sqrt { - \mu } \,\xi )}}} \right),\quad \mu < 0 \hfill \\ &F_{2} (\xi ) = \frac{{G^{\prime}}}{G} = \sqrt \mu \left( {\frac{ - A\,\sin (\sqrt \mu \,\xi ) + B\,\cos (\sqrt \mu \,\xi )}{A\,\cos (\sqrt \mu \,\xi ) + B\,\sin (\sqrt \mu \,\xi )}} \right),\quad \mu > 0 \hfill \\ \end{aligned}$$

where *A*, *B* are arbitrary constants.

Step 7: Assume that the constants *a*_*i*_, *b*_*i*_ (*i* = −*N*···*N*), *λ*, *V* can be obtained by solving the algebraic equations in Step 5. Substituting these constants and the solutions given in (6) into (4), we obtain wave solutions of Eq. (1) directly.

## Application of the method

In this section, the method discussed in “Description of the method” brings into look into wide-ranging and innovative solutions to the (1+1)-dimensional mBBM equation (Zayed and Al-Joudi 2010):

7 $$u_{t} + u_{x} - \alpha \,\,u^{2} \,u_{x} + u_{x\,x\,x} = 0,$$

where *α* is a non-zero positive constant. This equation was derived to describe an approximation for surface long waves in nonlinear dispersive media. It can also characterize the hydromagnetic waves in cold plasma, acoustic waves in inharmonic crystals and acoustic gravity waves in compressible fluids. We now examine solutions to (7) by using the proposed ansatz scheme. By means of the traveling wave transformation (2) and integrating once, we obtain

8 $$\left( {1 - V} \right)\,u - \frac{1}{3}\,\alpha \,u^{3} + u^{{\prime \prime }} + C = 0,$$

where primes indicate derivatives with respect to *ξ* and *C* is an integration constant to be determined. Using homogeneous balance between the highest order nonlinear term *u*^3^ and the linear term of the highest order *u*^″^ involved in (8) yields *N* = 1. Therefore, the suggested ansatz scheme allows us to set the solution of (8) as follows:

9 $$\begin{aligned} u(\xi ) &= a_{0} + \frac{{a_{1} \,(G^{\prime } /G)}}{{1 + \lambda \,(G^{\prime } /G)}} + \frac{{a_{ - 1} \,[1 + \lambda \,(G^{\prime } /G)]}}{{(G^{\prime } /G)}} \hfill \\ & \quad + b_{0} \,(G^{\prime } /G)^{ - 1} \sqrt {\sigma \,[1 + (G^{\prime } /G)^{2} /\mu ]} \\ & \quad + b_{1} \,\sqrt {\sigma \,[1 + (G^{\prime } /G)^{2} /\mu ]} + b_{ - 1} \,(G^{\prime } /G)^{ - 2} \,\sqrt {\sigma \,[1 + (G^{\prime } /G)^{2} /\mu ]} , \\ \end{aligned}$$

where *a*_0_, *a*_1_, *a*_−1_, *b*_0_, *b*_1_ and *b*_−1_ are constants to be determined and *G* = *G*(*ξ*) satisfies (5). Substituting (9) jointly with (5) into (8) and collecting each coefficient with the equal orders of (*G*^′^/*G*)^*j*^ and $(G^{\prime } /G)^{j} \,\sqrt {\sigma \,[1 + (G^{\prime } /G)^{2} /\mu ]}$, and setting them to zero yield an over-determined system of algebraic equations for *a*_0_, *a*_1_, *a*_−1_, *b*_0_, *b*_1_, *b*_−1_, *V*, *λ* and *C* (for minimalism the equations have not been displayed here). Solution of this over-determined system of algebraic equations with the help of computer algebra yields:

Case 1: *a*_1_ = 0, *a*_−1_ = 0, *a*_0_ = 0, *b*_0_ = *b*_−1_ = 0, $$b_{1} = \pm \sqrt {\frac{6\,\mu }{\sigma \,\alpha }}$$, *V* = 1 − *μ*, *λ* = *λ*, *C* = 0.

Case 2: $$a_{0} = \pm \frac{{\lambda \,\mu \,\sqrt {6\,\alpha } }}{\alpha }$$, $$a_{1} = \mp \sqrt {\frac{6}{\alpha }} \,\left( {1 + \lambda^{2} \,\mu } \right)$$, *a*_−1_ = 0, *b*_0_ = *b*_−1_ = 0, *b*_1_ = 0, *V* = 1 + 2*μ*, *λ* = *λ*, *C* = 0.

Case 3: $$a_{1} = \pm \sqrt {\frac{3}{2\,\alpha }}$$, *a*_−1_ = 0, $$b_{1} = \pm \sqrt {\frac{3\,\mu }{2\,\alpha \,\sigma }}$$, *a*_0_ = *b*_0_ = *b*_−1_ = 0, $$V = 1 + \,\frac{\mu }{2}$$, *λ* = 0, *C* = 0.

Case 4: $$a_{1} = \pm \sqrt {\frac{6}{\alpha }}$$, $$a_{ - 1} = \pm \sqrt {\frac{6}{\alpha }} \,\mu$$, *a*_0_ = *b*_0_ = *b*_1_ = *b*_−1_ = 0, *V* = 1 − 4*μ*, *λ* = 0, *C* = 0.

$$a_{1} = \pm \sqrt {\frac{6}{\alpha }}$$, $$a_{ - 1} = \mp \sqrt {\frac{6}{\alpha }} \,\mu$$, *a*_0_ = *b*_0_ = *b*_1_ = *b*_−1_ = 0, *V* = 1 + 8*μ*, *λ* = 0, *C* = 0.

Case 5: *a*_1_ = 0, $$a_{ - 1} = \mp \mu \,\sqrt {\frac{6}{\alpha }}$$, *b*_1_ = 0, *a*_0_ = *a*_0_, *b*_0_ = *b*_−1_ = 0, *V* = 1 + 2*μ*, $$\lambda = \pm \frac{{\alpha \,a_{0} }}{\mu }\sqrt {\frac{1}{6\,\alpha }}$$, *C* = 0.

Case 6: $$a_{1} = \mp \frac{{\left( {6\,\mu + \alpha \,a_{0}^{2} } \right)}}{\mu }\sqrt {\frac{1}{6\,\alpha }}$$, $$a_{ - 1} = \mp \mu \,\sqrt {\frac{6}{\alpha }}$$, *a*_0_ = *a*_0_, *b*_0_ = *b*_−1_ = *b*_1_ = 0, *V* = 1 − 4*μ* − *αa*_0_^2^, $$\lambda = \pm \frac{{\alpha \,a_{0} }}{\mu }\sqrt {\frac{1}{6\,\alpha }}$$, $$C = \frac{2}{3}\,(6\,\mu + \alpha \,a_{0}^{2} )\,a_{0}$$

Case 7: *a*_1_ = *a*_0_ = *a*_−1_ = *b*_1_ = *b*_−1_ = 0, $$b_{0} = \pm \mu \,\sqrt {\frac{6}{\,\sigma \,\alpha }}$$, *V* = 1 − *μ*, *λ* = *λ*, *C* = 0.

Case 8: $$a_{1} = \pm \sqrt {\frac{3}{2\,\alpha }}$$, $$a_{0} = \sqrt {\frac{3\,\mu }{2\,\alpha }}$$, $$a_{ - 1} = \pm \mu \sqrt {\frac{3}{2\,\alpha }}$$, $$b_{1} = \pm \sqrt {\frac{3\,\mu }{2\,\sigma \,\alpha }}$$, $$b_{0} = \mp \mu \sqrt {\frac{3}{2\,\sigma \,\alpha }}$$, *b*_−1_ = 0, $$V = 1 - \frac{5}{2}\mu$$, *λ* = 0,  $$C = - \mu \sqrt {\frac{6\,\mu }{\alpha }}$$

$$a_{1} = \pm \sqrt {\frac{3}{2\,\alpha }}$$, $$a_{0} = - \sqrt {\frac{3\,\mu }{2\,\alpha }}$$, $$a_{ - 1} = \pm \mu \sqrt {\frac{3}{2\,\alpha }}$$, $$b_{1} = \pm \sqrt {\frac{3\,\mu }{2\,\sigma \,\alpha }}$$, $$b_{0} = \mp \mu \sqrt {\frac{3}{2\,\sigma \,\alpha }}$$, *b*_−1_ = 0, $$V = 1 - \frac{5}{2}\mu$$, *λ* = 0, $$C = \mu \sqrt {\frac{6\,\mu }{\alpha }}$$.

Case 9: *a*_1_ = 0, $$a_{0} = - \lambda \,\mu \,\sqrt {\frac{3}{2\,\alpha }}$$, $$a_{ - 1} = \,\mu \,\sqrt {\frac{3}{2\,\alpha }}$$, *b*_1_ = 0, $$b_{0} = \pm \mu \,\sqrt {\frac{3}{2\,\sigma \,\alpha }}$$, *b*_−1_ = 0, $$V = 1 + \frac{1}{2}\mu$$, *λ* = *λ*, *C* = 0.

*a*_1_ = 0, $$a_{0} = \lambda \,\mu \,\sqrt {\frac{3}{2\,\alpha }}$$, $$a_{ - 1} = \, - \mu \,\sqrt {\frac{3}{2\,\alpha }}$$, *b*_1_ = 0, $$b_{0} = \pm \mu \,\sqrt {\frac{3}{2\,\sigma \,\alpha }}$$, *b*_−1_ = 0, $$V = 1 + \frac{1}{2}\mu$$, *λ* = *λ*, *C* = 0.

Zayed and Al-Joudi (2010) investigated traveling wave solutions to the modified Benjamin–Bona–Mahony equation by using the extended (*G*^′^/*G*)-expansion method and they achieved only three sets of solutions of the algebraic equations (only cases 1, 2 and 3 were obtained when *λ* = 0**)**. Using (6) into the solution (9) and substituting Cases 1–9, we obtain abundant traveling wave solutions including soliton, singular soliton, periodic solution, etc. to the modified Benjamin–Bona–Mahony equation in the following:

### Soliton solutions

When *μ* < 0, then Case 1 yields the following traveling wave solution:

10 $$u_{1,1} (\xi ) = \pm \sqrt {\frac{6\,\mu }{\sigma \,\alpha }} \,\,\sqrt {\sigma \left[ {1 - \left( {\frac{{A\,\sinh (\sqrt { - \mu } \,\xi ) + B\,\cosh (\sqrt { - \mu } \,\xi )}}{{A\,\cosh (\sqrt { - \mu } \,\xi ) + B\,\sinh (\sqrt { - \mu } \,\xi) }}} \right)^{2} } \right]} ,$$

where *ξ* = *x* − (1 − *μ*)*t* and *A*, *B* are arbitrary constants while *σ* = ±1.

### Particular cases

If we set specific values of *A* and *B*, various known solutions can be rediscovered. For example, soliton, periodic and complex solutions can be derived from the traveling wave solutions (10):

1. Soliton solutions

Setting *A* ≠ 0 but *B* = 0 in *u*_1,1_, we obtain

11a $$u_{1,2} (\xi ) = \pm \sqrt {\frac{6\,\mu }{\alpha }} \,\,\sec h( {\sqrt { - \mu } \,\xi }).$$

If *A* ≠ 0 and *A* > *B*, we obtain

11b $$u_{1,3} (\xi ) = \pm \sqrt {\frac{6\,\mu }{\alpha }} \,\,\sec h(\sqrt { - \mu } \,\xi + \varphi_{0} ),$$

where *φ*_0_ = tanh^−1^(*B*/*A*) and the value of *σ* is positive. These are exact bell-type soliton solutions.

2. Singular soliton solution

Setting *A* = 0 but *B* ≠ 0, we obtain

12 $$u_{1,4} (\xi ) = \pm \sqrt {\frac{ - 6\,\mu }{\alpha }} \,\,\cos ech(\sqrt { - \mu } \,\xi ).$$

Solution (12) is the singular soliton solution. Singular solitons are another kind of solitary waves that appear with a singularity, usually infinite discontinuity (Wazwaz 2012). Singular solitons can be connected to solitary waves when the center position of the solitary wave is imaginary (Drazin and Johnson 1989). Therefore it is not irrelevant to address the issue of singular solitons. This solution has spike and therefore it can probably provide an explanation to the formation of Rogue waves.

### Periodic solutions

When *μ* > 0, Case 1 yields the following periodic solution:

13 $$u_{1,5} (\xi ) = \pm \sqrt {\frac{6\,\mu }{\sigma \,\alpha }} \,\,\sqrt {\sigma \left[ {1 + \left( {\frac{ - A\,\sin (\sqrt \mu \,\xi ) + B\,\cos (\sqrt \mu \,\xi )}{A\,\cos (\sqrt \mu \,\xi ) + B\,\sin (\sqrt \mu \,\xi )}} \right)^{2} } \right]} .$$

When *A* and *B* receive particular values, different known solutions will be rediscovered. For example:

If *A* ≠ 0 but *B* = 0, we obtain

14 $$u_{1,6} (\xi ) = \pm \sqrt {\frac{6\,\mu }{\alpha }} \,\,\sec (\sqrt \mu \,\xi ).$$

When *A* ≠ 0 and *A* > *B*, we obtain

15 $$u_{1,7} (\xi ) = \pm \sqrt {\frac{6\,\,\mu }{\alpha }} \,\,\sec ( {\sqrt \mu \,\xi + \varphi_{0} } ),\,\quad \varphi_{0} = \tan^{ - 1} ({B \mathord{\left/ {\vphantom {B A}} \right.} A}).$$

If *A* = 0 but *B* ≠ 0, we obtain

16 $$u_{1,8} (\xi ) = \pm \sqrt {\frac{6\,\mu }{\alpha }} \,\,\cos ec(\sqrt \mu \,\xi ).$$

Periodic traveling waves play an important role in numerous physical phenomena, including reaction–diffusion–advection systems, impulsive systems, self-reinforcing systems, etc. Mathematical modelling of many intricate physical events, for instance biology, chemistry, physics, mathematical physics and many more phenomena resemble periodic traveling wave solutions.

Case 2 gives the following traveling wave solutions:

When *μ* < 0,

17 $$u_{2,1} (\xi ) = \pm \,\lambda \,\mu \,\sqrt {\frac{6}{\alpha }} \mp \sqrt {\frac{6}{\alpha }} \,\,\left[ {\frac{{(1 + \lambda^{2} \,\mu )\,F_{1} (\xi )}}{{1 + \lambda \,F_{1} (\xi )}}} \right].$$

When *μ* > 0,

18 $$u_{2,2} (\xi ) = \pm \,\lambda \,\mu \,\sqrt {\frac{6}{\alpha }} \mp \sqrt {\frac{6}{\alpha }} \,\,\left[ {\frac{{(1 + \lambda^{2} \,\mu )\,F_{2} (\xi )}}{{1 + \lambda \,F_{2} (\xi )}}} \right],\,$$

where *ξ* = *x* − (1 + 2*μ*)*t*.

Case 3 gives the following traveling wave solutions:

When *μ* < 0,

19 $$u_{3,1} (\xi ) = \pm \sqrt {\frac{3}{2\,\alpha }} \,F_{1} (\xi ) \pm \sqrt {\frac{3\,\mu }{2\,\,\alpha \,\sigma }} \,\,\sqrt {\sigma \left[ {1 + \frac{1}{\mu }(F_{1} (\xi ))^{2} } \right]} .$$

When *μ* > 0,

20 $$u_{3,2} (\xi ) = \pm \sqrt {\frac{3}{2\,\alpha }} \,F_{2} (\xi ) \pm \sqrt {\frac{3\,\mu }{2\,\,\alpha \,\sigma }} \,\,\sqrt {\sigma \left[ {1 + \frac{1}{\mu }(F_{2} (\xi ))^{2} } \right],}$$

where *ξ* = *x* − (1 + *μ*/2)*t*.

Case 4 gives the following traveling wave solutions:

When *μ* < 0,

21 $$u_{4,1} (\xi ) = \pm \sqrt {\frac{6}{\alpha }} \,F_{1} (\xi ) \pm \sqrt {\frac{6}{\alpha }} \,\mu \,(F_{1} (\xi ))^{ - 1} ,$$

and

22 $$u_{4,2} (\xi ) = \pm \sqrt {\frac{6}{\alpha }} \,F_{1} (\xi ) \mp \sqrt {\frac{6}{\alpha }} \,\mu \,(F_{1} (\xi ))^{ - 1} .$$

When *μ* > 0,

23 $$u_{4,3} (\xi ) = \pm \sqrt {\frac{6}{\alpha }} \,F_{2} (\xi ) \pm \sqrt {\frac{6}{\alpha }} \,\mu \,(F_{2} (\xi ))^{ - 1} ,$$

and

24 $$u_{4,4} (\xi ) = \pm \sqrt {\frac{6}{\alpha }} \,F_{2} (\xi ) \mp \sqrt {\frac{6}{\alpha }} \,\mu \,(F_{2} (\xi ))^{ - 1} ,$$

where *ξ* = *x* − (1 − 4*μ*)*t*.

Case 5 gives the following traveling wave solutions:

When *μ* < 0,

25 $$u_{5,1} (\xi ) = a_{0} \mp \sqrt {\frac{6}{\alpha }} \,\mu \,\left[ {1 \pm \frac{{a_{0} \,\alpha }}{\mu }\,\sqrt {\frac{1}{6\,\alpha }} \,F_{1} (\xi )} \right]\,(F_{1} (\xi ))^{ - 1} .$$

When *μ* > 0,

26 $$u_{5,2} (\xi ) = a_{0} \mp \sqrt {\frac{6}{\alpha }} \,\mu \,\left[ {1 \pm \frac{{a_{0} \,\alpha }}{\mu }\,\sqrt {\frac{1}{6\,\alpha }} \,F_{2} (\xi )} \right]\,(F_{2} (\xi ))^{ - 1} ,$$

where *ξ* = *x* − (1 + 2*μ*)*t*.

Case 6 gives the following traveling wave solutions:

When *μ* < 0,

27 $$u_{6,1} (\xi ) = a_{0} \mp \frac{{(6\,\mu + \alpha \,a_{0}^{2} )\,F_{1} (\xi )}}{{\mu \,\sqrt {6\,\alpha } \pm \alpha \,a_{0} \,F_{1} (\xi )}} \mp \sqrt {\frac{6}{\alpha }} \,\mu \,\left[ {1 \pm \frac{{a_{0} \,\alpha }}{\mu }\,\sqrt {\frac{1}{6\,\alpha }} \,F_{1} (\xi )} \right]\,(F_{1} (\xi ))^{ - 1} .$$

When *μ* > 0,

28 $$u_{6,2} (\xi ) = a_{0} \mp \frac{{(6\,\mu + \alpha \,a_{0}^{2} )\,F_{2} (\xi )}}{{\mu \,\sqrt {6\,\alpha } \pm \alpha \,a_{0} \,F_{2} (\xi )}} \mp \sqrt {\frac{6}{\alpha }} \,\mu \,\left[ {1 \pm \frac{{a_{0} \,\alpha }}{\mu }\,\sqrt {\frac{1}{6\,\alpha }} \,F_{2} (\xi )} \right]\,(F_{2} (\xi ))^{ - 1} ,$$

where *ξ* = *x* − (1 − 4*μ* − *a*_0_^2^*α*)*t*.

Case 7 gives the following traveling wave solutions:

When *μ* < 0,

29 $$u_{7,1} (\xi ) = \pm \mu \,\sqrt {\frac{6}{\sigma \,\alpha }} \,\,\sqrt {\sigma \left[ {1 + \frac{1}{\mu }F_{1}^{2} (\xi )} \right]} \,F_{1}^{ - 1} (\xi ).$$

When *μ* > 0,

30 $$u_{7,2} (\xi ) = \pm \mu \,\sqrt {\frac{6}{\sigma \,\alpha }} \,\,\sqrt {\sigma \left[ {1 + \frac{1}{\mu }F_{2}^{2} (\xi )} \right]} \,F_{2}^{ - 1} (\xi ),$$

where *ξ* = *x* − (1 − *μ*)*t*.

Case 8 gives the following exact traveling wave solutions:

When *μ* < 0,

31 $$\begin{aligned} u_{8,1} (\xi )& = \mp \,\varOmega \,\sqrt \mu \pm \varOmega \,\,F_{1} (\xi ) \pm \varOmega \,\mu \,\,(F_{1} (\xi ))^{ - 1} \pm \varOmega \,\mu \,\sqrt {\frac{1}{\sigma }} \,(F_{1} (\xi ))^{ - 1}\\ & \quad \times \sqrt {\sigma \,\left[ {1 + \frac{1}{\mu }(F_{1} (\xi ))^{2} } \right]} + \varOmega \,\sqrt {\frac{\mu }{\sigma }} \, \sqrt {\sigma \,\left[ {1 + \frac{1}{\mu }(F_{1} (\xi ))^{2} } \right]} , \\ \end{aligned}$$

32 $$\begin{aligned} u_{8,2} (\xi ) &= \mp \,\,\varOmega \,\sqrt \mu \pm \varOmega \,\,F_{1} (\xi ) \pm \varOmega \,\mu \,\,(F_{1} (\xi ))^{ - 1} \mp \varOmega \,\mu \,\sqrt {\frac{1}{\sigma }} \,(F_{1} (\xi ))^{ - 1} \\ & \quad \times \sqrt {\sigma \,\left[ {1 + \frac{1}{\mu }(F_{1} (\xi ))^{2} } \right]} \quad - \varOmega \,\sqrt {\frac{\mu }{\sigma }} \,\sqrt {\sigma \,\left[ {1 + \frac{1}{\mu }(F_{1} (\xi ))^{2} } \right]} . \\ \end{aligned}$$

When *μ* > 0,

33 $$\begin{aligned} u_{8,3} (\xi ) &= \mp \,\varOmega \,\sqrt \mu \pm \varOmega \,F_{2} (\xi ) \pm \varOmega \,\mu \,\,(F_{2} (\xi ))^{ - 1} \pm \varOmega \,\mu \,\sqrt {\frac{1}{\sigma }} \,(F_{2} (\xi ))^{ - 1} \\ & \quad \times \sqrt {\sigma \,\left[ {1 + \frac{1}{\mu }(F_{2} (\xi ))^{2} } \right]} + \varOmega \,\sqrt {\frac{\mu }{\sigma }} \,\sqrt {\sigma \,\left[ {1 + \frac{1}{\mu }(F_{2} (\xi ))^{2} } \right]} , \\ \end{aligned}$$

34 $$\begin{aligned} u_{8,4} (\xi )& = \, \mp \varOmega \,\sqrt \mu \pm \varOmega \,\,F_{2} (\xi ) \pm \varOmega \,\mu \,\,(F_{2} (\xi ))^{ - 1} \mp \varOmega \,\mu \,\sqrt {\frac{1}{\sigma }} \,(F_{2} (\xi ))^{ - 1} \\ & \quad \times \sqrt {\sigma \,\left[ {1 + \frac{1}{\mu }(F_{2} (\xi ))^{2} } \right]} - \varOmega \,\sqrt {\frac{\mu }{\sigma }} \,\sqrt {\sigma \,\left[ {1 + \frac{1}{\mu }(F_{2} (\xi ))^{2} } \right]} , \\ \end{aligned}$$

where *ξ* = *x* − (1 − 5*μ*/2)*t* and $$\varOmega = \sqrt {\frac{3}{2\,\alpha }}$$.

Case 9 gives the following exact traveling wave solutions:

When *μ* < 0,

35 $$\begin{aligned} &u_{9,1} (\xi ) = - \varOmega \,\lambda \,\mu \, + \, \varOmega \,\mu \,\,[1\, +\, \lambda \,F_{1} (\xi )]\,\,(F_{1} (\xi ))^{ - 1} \pm \,\varOmega \,\mu \,\sqrt {\frac{1}{\sigma }} \,(F_{1} (\xi ))^{ - 1} \\ & \quad \times \sqrt {\sigma \,\left[ {1 + \frac{1}{\mu }(F_{1} (\xi ))^{2} } \right]}, \end{aligned}$$

36 $$\begin{aligned} &u_{9,2} (\xi ) = \varOmega \,\lambda \,\mu \, - \varOmega \,\mu \,\,[1 + \lambda \,F_{1} (\xi )]\,\,(F_{1} (\xi ))^{ - 1} \pm \varOmega \,\mu \,\sqrt {\frac{1}{\sigma }} \,(F_{1} (\xi ))^{ - 1} \\ & \quad \times \sqrt {\sigma \,\left[ {1 + \frac{1}{\mu }(F_{1} (\xi ))^{2} } \right]} . \end{aligned}$$

When *μ* > 0,

37 $$\begin{aligned} &u_{9,3} (\xi ) = - \varOmega \,\lambda \,\mu \, + \varOmega \,\mu \,\,[1 + \lambda \,F_{2} (\xi )]\,\,(F_{2} (\xi ))^{ - 1} \pm \varOmega \,\mu \,\sqrt {\frac{1}{\sigma }} \,(F_{2} (\xi ))^{ - 1} \\ & \quad \times \sqrt {\sigma \,\left[ {1 + \frac{1}{\mu }(F_{2} (\xi ))^{2} } \right]} , \end{aligned}$$

38 $$\begin{aligned} &u_{9,4} (\xi ) = \varOmega \,\lambda \,\mu \, - \varOmega \,\mu \,\,[1 + \lambda \,F_{2} (\xi )]\,\,(F_{2} (\xi ))^{ - 1} \pm \varOmega \,\mu \,\sqrt {\frac{1}{\sigma }} \,(F_{2} (\xi ))^{ - 1} \\ & \quad \times \sqrt {\sigma \,\left[ {1 + \frac{1}{\mu }(F_{2} (\xi ))^{2} } \right]} , \end{aligned}$$

where *ξ* = *x* − (1 + *μ*/2)*t* and $$\varOmega = \sqrt {\frac{3}{2\,\alpha }}$$.

Substitution of the values of *F*_1_(*ξ*) and *F*_2_(*ξ*) into solutions (17–38) yields further general solutions to the modified Benjamin-Bona-Mahony equation. Setting particular values of the free parameters involved in solutions (10–38) abundant soliton, singular solitons, periodic solutions and general solitary wave solutions can be found.

## Results and discussion

Zayed and Al-Joudi (2010) examined the solutions to the mBBM equation by using the extended (*G*^′^/*G*)-expansion method and obtained only six solutions [solutions (3.9–3.14), see (Zayed and Al-Joudi 2010) for details]. On the other hand by means of the new ansatz of the (*G*^′^/*G*)-expansion, we obtain 24 solutions. It is noteworthy to refer that some of our obtained solutions are identical to the solutions achieved by Zayed and Al-Joudi (2010) which validate our solutions and some are new. A comparison between the obtained solutions in this article and Zayed and Al-Joudi’s (2010) solutions are given in the following Table 1.

**Table 1:** Comparison between Zayed and Al-Joudi’s solutions and solutions obtained in this article

Solutions obtained in this article	Zayed and Al-Joudi (2010) solutions
If *μ* < 0, case 1 yields $$\begin{aligned} u_{1,1} (\xi ) &= \pm \sqrt {\frac{6\,\mu }{\sigma \,\alpha }} \, \hfill \\ & \quad \times \,\sqrt {\sigma \left[ {1 - \,\left( {\frac{{A\,\sinh (\sqrt { - \mu } \,\xi ) + B\,\cosh (\sqrt { - \mu } \,\xi )}}{{A\,\cosh (\sqrt { - \mu } \,\xi ) + B\,\sinh (\sqrt { - \mu } \,\xi )}}} \right)^{2} } \right]} \hfill \\ \end{aligned}$$	If *σ* = ± 1 and *μ* < 0 solution (3.10) becomes $$\begin{aligned} u(\xi ) & = \pm \sqrt {\frac{6\,\mu }{\sigma \,\alpha }} \hfill \\ & \quad \times \,\,\sqrt {\sigma \left[ {1 - \,\left( {\frac{{A\,\sinh (\sqrt { - \mu } \,\xi ) + B\,\cosh (\sqrt { - \mu } \,\xi )}}{{A\,\cosh (\sqrt { - \mu } \,\xi ) + B\,\sinh (\sqrt { - \mu } \,\xi )}}} \right)^{2} } \right]} \hfill \\ \end{aligned}$$
If *λ* = 0 and *μ* < 0, case 2 yields $u_{2,1} (\xi ) = \pm \sqrt {\frac{ - 6\,\mu }{\alpha }} \,\left( {\frac{{A\,\sinh (\sqrt { - \mu } \,\xi ) + B\,\cosh (\sqrt { - \mu } \,\xi )}}{{A\,\cosh (\sqrt { - \mu } \,\xi ) + B\,\sinh (\sqrt { - \mu } \,\xi )}}} \right)$	If *σ* = ±1 and *μ* < 0 solution (3.9) becomes $u(\xi ) = \pm \sqrt {\frac{ - 6\,\mu }{\alpha }} \,\left( {\frac{{A\,\sinh (\sqrt { - \mu } \,\xi ) + B\,\cosh (\sqrt { - \mu } \,\xi )}}{{A\,\cosh (\sqrt { - \mu } \,\xi ) + B\,\sinh (\sqrt { - \mu } \,\xi )}}} \right)$
If *μ* < 0, Case 3 yields $$\begin{aligned} u_{3,1} (\xi ) = & \pm \sqrt {\frac{3}{2\,\alpha }} \,\left\{ {\sqrt \mu \,\sqrt {\,\left[ {1 - \left( {\frac{\begin{aligned} A\,\sinh (\sqrt { - \mu } \,\xi ) \hfill \\ + B\,\cosh (\sqrt { - \mu } \,\xi ) \hfill \\ \end{aligned} }{\begin{aligned} A\,\cosh (\sqrt { - \mu } \,\xi ) \hfill \\ + B\,\sinh (\sqrt { - \mu } \,\xi ) \hfill \\ \end{aligned} }} \right)^{2} } \right]} } \right. \hfill \\ & \quad + \left. {\sqrt { - \mu } \,\left( {\frac{{A\,\sinh (\sqrt { - \mu } \,\xi ) + B\,\cosh (\sqrt { - \mu } \,\xi )}}{{A\,\cosh (\sqrt { - \mu } \,\xi ) + B\,\sinh (\sqrt { - \mu } \,\xi )}}} \right)} \right\} \hfill \\ \end{aligned}$$	If *σ* = ±1 and *μ* < 0 solution (3.11) becomes $$\begin{aligned} u(\xi ) = \pm \sqrt {\frac{3}{2\,\alpha }} \,\left\{ {\sqrt \mu \,\sqrt {\,\left[ {1 - \left( {\frac{\begin{aligned} A\,\sinh (\sqrt { - \mu } \,\xi ) \hfill \\ + B\,\cosh (\sqrt { - \mu } \,\xi ) \hfill \\ \end{aligned} }{\begin{aligned} A\,\cosh (\sqrt { - \mu } \,\xi ) \hfill \\ + B\,\sinh (\sqrt { - \mu } \,\xi ) \hfill \\ \end{aligned} }} \right)^{2} } \right]} } \right. \hfill \\ + \left. {\sqrt { - \mu } \,\left( {\frac{{A\,\sinh (\sqrt { - \mu } \,\xi ) + B\,\cosh (\sqrt { - \mu } \,\xi )}}{{A\,\cosh (\sqrt { - \mu } \,\xi ) + B\,\sinh (\sqrt { - \mu } \,\xi )}}} \right)} \right\} \hfill \\ \end{aligned}$$
When *μ* > 0, case 1 yields $$\begin{aligned} u_{1,5} (\xi ) = \pm \sqrt {\frac{6\,\mu }{\sigma \,\alpha }} \, \hfill \\ \times \,\sqrt {\sigma \left[ {1 - \,\left( {\frac{ - A\,\sin (\sqrt \mu \,\xi ) + B\,\cos (\sqrt \mu \,\xi )}{A\,\cos (\sqrt \mu \,\xi ) + B\,\sin (\sqrt \mu \,\xi )}} \right)^{2} } \right]} \hfill \\ \end{aligned}$$	If *σ* = ±1 and *μ* > 0 solution (3.13) becomes $$\begin{aligned} u(\xi ) = \pm \sqrt {\frac{6\,\mu }{\sigma \,\alpha }} \, \hfill \\ \times \,\sqrt {\sigma \left[ {1 - \,\left( {\frac{{ - A\,\sin \left( {\sqrt \mu \,\xi } \right) + B\,\cos \left( {\sqrt \mu \,\xi } \right)}}{{A\,\cos \left( {\sqrt \mu \,\xi } \right) + B\,\sin \left( {\sqrt \mu \,\xi } \right)}}} \right)^{2} } \right]} \hfill \\ \end{aligned}$$
If *λ* = 0 and *μ* > 0, case 2 yields $u_{2,2} (\xi ) = \pm \sqrt {\frac{6\,\mu }{\alpha }} \,\left( {\frac{ - A\,\sin (\sqrt \mu \,\xi ) + B\,\cos (\sqrt \mu \,\xi )}{A\,\cos (\sqrt \mu \,\xi ) + B\,\sin (\sqrt \mu \,\xi )}} \right)$	If *σ* = ±1 and *μ* > 0 solution (3.12) becomes $u(\xi ) = \pm \sqrt {\frac{6\,\mu }{\alpha }} \,\left( {\frac{ - A\,\sin (\sqrt \mu \,\xi ) + B\,\cos (\sqrt \mu \,\xi )}{A\,\cos (\sqrt \mu \,\xi ) + B\,\sin (\sqrt \mu \,\xi )}} \right)$
If *μ* > 0, case 3 yields $$\begin{aligned} u_{3,2} (\xi ) = \pm \sqrt {\frac{3}{2\,\alpha }} \,\left\{ {\sqrt \mu \,\sqrt {\,\left[ {1 - \left( {\frac{\begin{aligned} - A\,\sin (\sqrt \mu \,\xi ) \hfill \\ + B\,\cos (\sqrt \mu \,\xi ) \hfill \\ \end{aligned} }{\begin{aligned} A\,\cos (\sqrt \mu \,\xi ) \hfill \\ + B\,\sin (\sqrt \mu \,\xi ) \hfill \\ \end{aligned} }} \right)^{2} } \right]} } \right. \hfill \\ + \left. {\sqrt \mu \,\left( {\frac{ - A\,\sin (\sqrt \mu \,\xi ) + B\,\cos (\sqrt \mu \,\xi )}{A\,\cos (\sqrt \mu \,\xi ) + B\,\sin (\sqrt \mu \,\xi )}} \right)} \right\} \hfill \\ \end{aligned}$$	If *σ* = ±1 and *μ* > 0 solution (3.14) becomes $$\begin{aligned} u(\xi ) = \pm \sqrt {\frac{3}{2\,\alpha }} \,\left\{ {\sqrt \mu \,\sqrt {\,\left[ {1 - \left( {\frac{\begin{aligned} - A\,\sin (\sqrt \mu \,\xi ) \hfill \\ + B\,\cos (\sqrt \mu \,\xi ) \hfill \\ \end{aligned} }{\begin{aligned} A\,\cos (\sqrt \mu \,\xi ) \hfill \\ + B\,\sin (\sqrt \mu \,\xi ) \hfill \\ \end{aligned} }} \right)^{2} } \right]} } \right. \hfill \\ + \left. {\sqrt \mu \,\left( {\frac{ - A\,\sin (\sqrt \mu \,\xi ) + B\,\cos (\sqrt \mu \,\xi )}{A\,\cos (\sqrt \mu \,\xi ) + B\,\sin (\sqrt \mu \,\xi )}} \right)} \right\} \hfill \\ \end{aligned}$$

Beyond the solutions specified in the Table 1 above, we obtain further new exact traveling wave solutions (21–38) which are not reported in the previous literature. These solutions might be much important for the explanation of some special physical phenomena. The solitary wave ansatz method can be applied to high-dimensional or coupled nonlinear PDEs in mathematical physics. We expect the attained solutions may be useful for further numerical analysis and may help the researchers to explain complex physical phenomena.

## Conclusion

Based on some existing extensions of the (*G*^′^/*G*)-expansion method, a new ansatz has been established to search for traveling wave solutions to nonlinear partial differential equations. The ansatz constructs abundant traveling wave solutions to the (1+1)-dimensional modified Benjamin–Bona–Mahony equation which illustrates the validity and effectiveness of the algorithm. The solutions obtained in this article are in more general forms and many known solutions to this equation are only special cases. This study shows that the proposed ansatz is reliable, effective and computerized which permit us to carry out complicated and tiresome algebraic calculation and giving new solutions to the applied equation. This ansatz can be applied to both single equation and coupled equations to establish further new solutions for other kinds of nonlinear partial differential equations.
